# Two-year clinical outcome of patients with mildly reduced ejection fraction after acute myocardial infarction: insights from the prospective KAMIR-NIH Registry

**DOI:** 10.3389/fcvm.2024.1458740

**Published:** 2024-09-20

**Authors:** Ho Sung Jeon, Jun-Won Lee, Jin Sil Moon, Dae Ryong Kang, Jung-Hee Lee, Young Jin Youn, Min-Soo Ahn, Sung Gyun Ahn, Byung-Su Yoo

**Affiliations:** ^1^ Division of Cardiology, Department of Internal Medicine, Yonsei University Wonju Severance Christian Hospital, Wonju, Republic of Korea; ^2^ Center of Biomedical Data Science, Yonsei University Wonju Severance Christian Hospital, Wonju, Republic of Korea

**Keywords:** acute myocardial infarction, mildly reduced ejection fraction, prognosis, prospective registry, left ventricular ejection fraction

## Abstract

**Background:**

Left ventricular ejection fraction (LVEF) is a crucial prognostic indicator of acute myocardial infarction (AMI). However, there is a lack of studies on the clinical characteristics and prognosis of patients with mildly reduced ejection fraction (EF) after AMI.

**Methods:**

We categorized 6,553 patients with AMI from the Korea Acute Myocardial Infarction Registry-National Institutes of Health (KAMIR-NIH) between November 2011 and December 2015 into three groups based on their EF, as assessed by echocardiography during index hospitalization: reduced EF (LVEF ≤40%), mildly reduced EF (LVEF 41%–49%), and preserved EF (LVEF ≥50%). The primary outcome was all-cause death within 2 years. The secondary outcomes included myocardial infarction (MI), revascularization, and patient-oriented composite endpoint (POCE), which was defined as a composite of all-cause death, any MI, or revascularization.

**Results:**

Of the total 6,553 patients, 884 (13.5%) were classified into the reduced EF group, 1,749 (26.7%) into the mildly reduced EF group, and 3,920 (59.8%) into the preserved EF group. Patients with mildly reduced EF exhibited intermediate mortality (reduced EF, 24.7%; mildly reduced EF, 8.3%; preserved EF, 4.6%; *p* < 0.0001), MI (3.9% vs. 2.7% vs. 2.6%; *p* < 0.0046), and POCE (33.0% vs. 15.6% vs. 12.4%; *p* < 0.0001) rates, albeit closer to those of the preserved EF. After adjustment for demographics, risk factors, admission status, and discharge medications, patients with mildly reduced EF showed a lower risk of all-cause death than those with reduced EF (mildly reduced EF group as a reference: HR, 1.74; 95% CI, 1.40–2.18; *p* < 0.001), but it did not differ significantly from those with preserved EF (HR, 0.94; 95% CI, 0.75–1.18; *p* = 0.999)

**Conclusions:**

Over a 2-year follow-up period, patients with AMI and mildly reduced EF demonstrated better prognoses than those with reduced EF, but did not differ significantly from those with preserved EF.

**Clinical Trial Registration:**

cris.nih.go.kr, identifier: KCT−0000863.

## Introduction

Ischemic heart disease (IHD), specifically acute myocardial infarction (AMI), is the leading cause of cardiovascular death and a significant contributor to heart failure (HF) (1, 2). After an episode of AMI, left ventricular ejection fraction (LVEF) reflects cardiac remodeling, infarct size, and prognosis (3). The prevalence of HF following AMI is a crucial clinical and public health issue not only due to its frequency (4, 5) but also due to its significant correlation with mortality (6, 7).

The current HF guidelines state that patients with HF and an LVEF of 41%–49% have HF with mid-range ejection fraction (EF) (8, 9). Subsequent studies have shown that patients with HF with mid-range EF have characteristics that fall between heart failure with reduced EF (HFrEF) and heart failure with preserved EF (HFpEF) (10). HF with mid-range EF is thought to be similar to HFrEF in terms of etiology and treatment response (11). The prevalence of IHD is similar between HF with mid-range EF and HFrEF groups, which is greater than that observed in the HFpEF group (12, 13). Patients with HF with mid-range EF or HFpEF have a lower cardiovascular risk than those with HFrEF. On the other hand, the risk of non-cardiovascular events is comparable or higher in those with HF with mid-range EF or HFpEF than in those with HFrEF (14, 15). This is believed to be due to the high prevalence of comorbidities in patients with HFpEF (16). Certain medical treatments used for HFrEF can also be beneficial for HF with mid-range EF (17, 18). Recently, the term “mid-range” was changed to “mildly reduced” and is currently used in the literature (11, 19, 20).

However, as HF is a clinical syndrome with various phenotypes, the prognosis may differ according to each phenotype (21). In particular, patients with mildly reduced EF after AMI may exhibit distinct characteristics compared to those with HFmrEF. However, research on the clinical characteristics and prognosis of these patients is insufficient. Therefore, we investigated the clinical features and prognosis of patients with mildly reduced EF after AMI.

## Methods

### Study population

**Figure 1:**
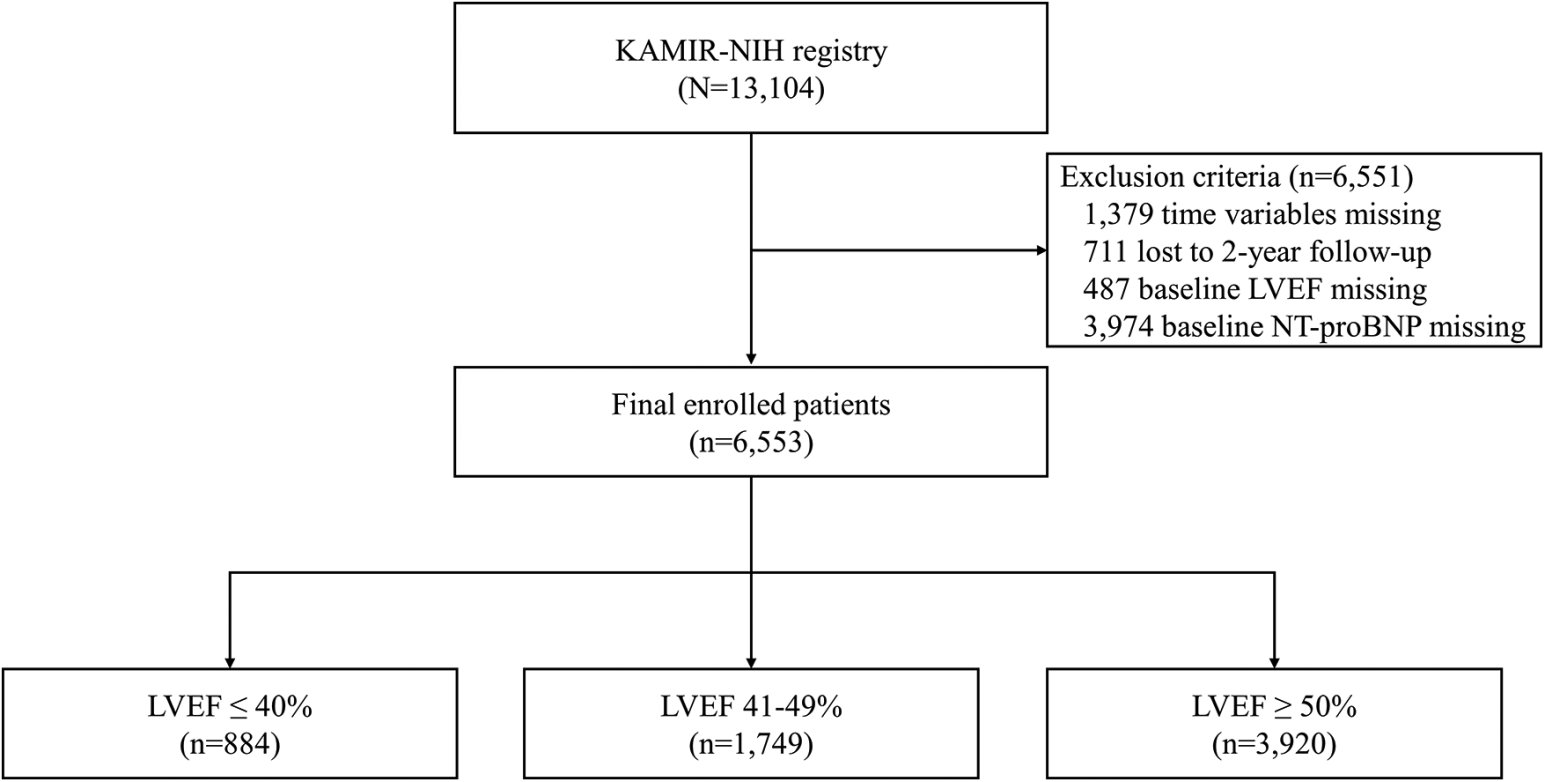
Study flowchart. KAMIR-NIH, Korea Acute Myocardial Infarction-National Institutes of Health; LVEF, left ventricular ejection fraction; NT-proBNP, N-terminal pro-B-type natriuretic peptide.

The Korean Acute Myocardial Infarction Registry-National Institutes of Health (KAMIR-NIH) is a prospective, multicenter, nationwide observational cohort study that enrolled patients diagnosed with AMI in 20 tertiary hospitals from November 2011 to December 2015 (22). The study protocol was approved by the Institutional Review Board at each participating hospital, and patients provided written informed consent to participate. Of the initial 13,104 patients, 6,553 patients were included in the study after excluding 3,974 individuals with missing N-terminal pro-B-type natriuretic peptide (NT-proBNP) values, 1,379 with missing time variable values, 711 who were lost to 2-year follow-up, and 487 with unmeasured EF (Figure 1). During the index hospitalization, the patients underwent echocardiography and were classified into three groups based on their LVEF: reduced (LVEF ≤40%), mildly reduced (LVEF 41%–49%), and preserved EF (LVEF ≥50%).

### Definitions

The definition of AMI was based on the acute myocardial injury, a rise and/or fall of cardiac troponin values with at least 1 value above the 99th percentile upper reference limit, and at least 1 of the following: presentation of chest pain; consecutive electrocardiogram (ECG) changes suggesting myocardial infarction (MI); development of pathologic Q waves; imaging evidence about an ischemic etiology; and identification of a coronary thrombus by angiography (23). ST-elevation myocardial infarction (STEMI) was defined as new ST-segment elevation in more than two contiguous leads, measuring >0.2 mV in leads V1−3 or 0.1 mV in other leads, or new left bundle branch block on a 12-lead ECG with at least one positive finding of cardiac troponin T or I. Non-ST-elevation MI (NSTEMI), on the other hand, was defined as at least one positive biomarker without ST-segment elevation. Chronic kidney disease (CKD) was defined as an estimated glomerular filtration rate (eGFR) of <60 ml/min/1.73 m^2^.

Experienced imaging cardiologists who were blinded to the clinical data performed echocardiography using a Vivid Ultrasound Systems (General Electric Medical System, Horten, Norway) or EPIQ (Philips Healthcare, Andover, MA, USA). Quantitative calculation using the modified Simpson's biplane method was recommended for LVEF measurement, but LVEF estimation by M-mode was also accepted.

Medical personnel and trained coordinators entered data using a web-based case report from the Internet-based Clinical Research and Trial Management System (iCReaT), a data management system established by the Centers for Disease Control and Prevention, Ministry of Health and Welfare, Republic of Korea (iCReaT Study No. C110016; cris.nih.go.kr, identifier: KCT-0000863). The demographic and basic characteristics, coronary angiography information, procedure-related information, echocardiography results, and drug treatment data were recorded. Treatment strategies and drug prescriptions were based on the decisions of healthcare providers. The patient was treated according to the latest guidelines. Patients were followed up either through outpatient visits or telephone interviews.

### Study outcomes

The primary outcome was 2-year all-cause death. The secondary outcomes included MI, revascularization, and patient-oriented composite endpoint (POCE), which was defined as all-cause death, any MI, or revascularization. All-cause death included all deaths related and unrelated to heart diseases. MI was defined as cardiac enzyme levels exceeding the upper limit of normal with ischemic symptoms or ECG changes irrespective of the previously treated coronary vessels. Revascularization was defined as the reperfusion of previously treated and other vessels. The staged manner of procedure was excluded from the revascularization endpoint.

### Statistical analysis

Data were presented as frequency and percentage, mean ± standard deviation (SD), or median with an interquartile range (IQR). To compare LVEF among the three groups, dichotomous variables were analyzed using Fisher's exact test or the Kruskal–Wallis test, and continuous variables were analyzed using one-way ANOVA. Two-year clinical evaluation indicators were compared using Kaplan–Meier analysis, and statistical significance was confirmed using the log-rank test for comparison between groups. Hazard ratios (HRs) were analyzed using Cox proportional hazards regression analysis. Adjusted HRs were calculated using Cox regression analysis with age, sex, clinical risk factors [diabetes mellitus (DM) and CKD], admission status (STEMI, heart rate, NT-proBNP, and hemoglobin), and discharge medications [beta-blockers, renin–angiotensin system (RAS) inhibitors, and statins]. The statistical analysis software SAS (version 9.4; SAS Institute, Cary, NC, USA) was used. A *p-*value of <0.05 was defined as statistically significant.

## Results

### Baseline clinical characteristics

The baseline characteristics of the patients are shown in Table 1. Of the total 6,553 patients, 884 (13.5%) were classified into the reduced EF group, 1,749 (26.7%) into the mildly reduced EF group, and 3,920 (59.8%) into the preserved EF group. The median EF (%) was 33 (IQR, 28–37), 46 (IQR, 43–48), and 58 (IQR, 54–62) in each respective group. The patients with mildly reduced EF had intermediate characteristics of age, sex, and body mass index (BMI), falling on the spectrum from reduced to preserved EF. Regarding clinical risk factors, the patients with mildly reduced EF had the lowest prevalence of HTN (reduced EF, 54.0%; mildly reduced EF, 46.9%; preserved EF, 49.3%; *p* = 0.0028), while the mildly reduced EF group had intermediate prevalence rates of DM (40.8% vs. 27.3% vs. 25.5%; *p* < 0.0001) and CKD (42.0% vs. 22.9% vs. 17.2%; *p* < 0.0001). The mildly reduced EF groups also had intermediate rates of previous MI (12.2% vs. 8.6% vs. 4.9%; *p* < 0.0001) and cerebrovascular accidents (CVA) (9.8% vs. 6.9% vs. 5.4%; *p* < 0.0001). The rate of clinical diagnosis of STEMI was the highest in the patients with mildly reduced EF (57.1% vs. 64.1% vs. 46.8%; *p* < 0.0001). The rate of blood pressure (BP) was comparable between the patients with mildly reduced EF and preserved EF groups but was lowest in those with reduced EF group [systolic blood pressure (mmHg), 120 vs. 130 vs. 130; *p* < 0.0001; diastolic blood pressure (mmHg), 78 vs. 80 vs. 80; *p* < 0.0001]. However, the mildly reduced EF group had an intermediate heart rate (beats/min) (88 vs. 80 vs. 74; *p* < 0.0001). In terms of laboratory values, the NT-proBNP level was intermediate but close to that in the preserved EF group (2,639.5 vs. 396.1 vs. 167.0; *p* < 0.0001), and hemoglobin showed a similar trend (13.2 vs. 14.2 vs. 14.3; *p* < 0.0001). The patients with Killip class ≥II comprised an intermediate proportion (47.9% vs. 24.8% vs. 15.6%; *p* < 0.0001). At the time of discharge, the prescription rates for aspirin and P2Y12 inhibitors were nearly 100% in all three groups. However, the prescription rates for beta-blockers, RAS inhibitors, and statins were lower in the reduced EF group compared to other groups.

**Table 1:** Baseline characteristics.

	Total (*N* = 6,553)	Reduced EF (*n* = 884)	Mildly reduced EF (*n* = 1,749)	Preserved EF (*n* = 3,920)	*p*-value
Age (year)	64 (54–73)	70 (59–77)	65 (54–74)	62 (54–72)	<0.0001
Male	4,897 (74.7)	602 (68.1)	1,286 (73.5)	3,009 (76.8)	<0.0001
Height (cm)	166 (160–170)	165 (157–170)	165 (159–170)	167 (160–171)	<0.0001
Weight (kg)	65 (58–73)	62 (54–70)	65 (56–72)	66 (59–74)	<0.0001
BMI (kg/m^2^)	23.9 (22.0–25.9)	23.2 (20.8–25.2)	23.7 (21.8–25.7)	24.1 (22.2–26.1)	<0.0001
Risk factors					
Hypertension	3,230 (49.3)	477 (54.0)	820 (46.9)	1,933 (49.3)	0.0028
DM	1,837 (28.0)	361 (40.8)	477 (27.3)	999 (25.5)	<0.0001
CKD	1,448 (22.1)	371 (42.0)	401 (22.9)	676 (17.2)	<0.0001
Dyslipidemia	673 (10.3)	71 (8.0)	156 (8.9)	446 (11.4)	0.0012
Previous MI	452 (6.9)	108 (12.2)	151 (8.6)	193 (4.9)	<0.0001
Old CVA	420 (6.4)	87 (9.8)	121 (6.9)	212 (5.4)	<0.0001
Current smoker	2,655 (40.5)	292 (33.0)	715 (40.9)	1,648 (42.0)	<0.0001
Associated dyspnea	1,457 (22.2)	353 (39.9)	416 (23.8)	688 (17.6)	<0.0001
Clinical diagnosis					<0.0001
STEMI	3,462 (52.8)	505 (57.1)	1,121 (64.1)	1,836 (46.8)	
NSTEMI	3,091 (47.2)	379 (42.9)	628 (35.9)	2,084 (53.2)	
Systolic BP (mmHg)	130 (110–150)	120 (106–140)	130 (110–150)	130 (114–150)	<0.0001
Diastolic BP (mmHg)	80 (70–90)	78 (66–90)	80 (70–90)	80 (70–90)	<0.0001
Heart rate (beats/minute)	77 (66–88)	88 (75–103)	80 (69–90)	74 (64–85)	<0.0001
NT-proBNP	263.1 (62.2–131.0)	2639.5 (330.2–8,324.0)	396.1 (74.7–1,722.0)	167.0 (49.3–679.3)	<0.0001
Hemoglobin (g/dl)	14.1 (12.6–15.3)	13.2 (11.5–14.6)	14.2 (12.7–15.4)	14.3 (12.9–15.4)	<0.0001
Glucose (mg/dl)	147 (120–196)	169 (131–245)	151 (124–198)	142 (117–186)	<0.0001
Creatinine (mg/dl)	0.9 (0.7–1.1)	1.0 (0.8–1.4)	0.9 (0.7–1.1)	0.9 (0.7–1.1)	<0.0001
Peak CK-MB (ng/ml)	51.6 (9.5–177.5)	57.3 (10.4–240.4)	94.8 (16.2–259.7)	39.4 (7.8–134.8)	<0.0001
Peak troponin I (ng/ml)	20.1 (3.4–50.8)	25.0 (4.6–97.9)	25.5 (6.7–80.1)	14.6 (2.6–38.3)	<0.0001
Total cholesterol (mg/dl)	177 (149–208)	167 (138–199)	177 (149–209)	179 (152–209)	<0.0001
Triglyceride (mg/dl)	105 (71–162)	91 (64–135)	102 (70–156)	110 (74–170)	<0.0001
HDL cholesterol (mg/dl)	41 (35–49)	41 (34–48)	42 (36–49)	41 (35–48)	0.0321
LDL cholesterol (mg/dl)	112 (86–137)	105 (76–131)	112 (85–138)	114 (89–138)	<0.0001
Killip class					<0.0001
Ⅰ	5,083 (77.6)	461 (52.2)	1,315 (75.2)	3,307 (84.4)	
Ⅱ	649 (9.9)	127 (14.4)	218 (12.5)	304 (7.8)	
Ⅲ	530 (8.1)	214 (24.2)	151 (8.6)	165 (4.2)	
Ⅳ	291 (4.4)	82 (9.3)	65 (3.7)	144 (3.7)	
Killip class ≥Ⅱ	1,470 (22.4)	423 (47.9)	434 (24.8)	613 (15.6)	<0.0001
LVEF,%	52 (45–59)	33 (28–37)	46 (43–48)	58 (54–62)	<0.0001
STD time (minute)	209 (87–717)	300 (120–1,391)	221 (93–673)	92 (79–631)	<0.0001
DTB time (minute)	90 (57–861)	85 (57–801)	75 (54–383)	136 (59–1,003)	<0.0001
Discharge medication					
Aspirin	6,544 (99.9)	883 (99.9)	1,747 (99.9)	3,914 (99.9)	1.0000^a^
P2Y12 inhibitor	6,532 (99.7)	883 (99.9)	1,743 (99.7)	3,906 (99.6)	0.5009
CCB	354 (5.4)	24 (2.7)	72 (4.1)	258 (6.6)	<0.0001
Beta-blocker	561 (85.6)	673 (76.1)	1,530 (87.5)	3,408 (86.9)	<0.0001
RAS inhibitor	5,287 (80.7)	630 (71.3)	1,389 (79.4)	3,268 (83.4)	<0.0001
Statin	6,107 (93.2)	730 (82.6)	1,622 (92.7)	3,755 (95.8)	<0.0001

Values are *n* (%) or medians (interquartile ranges). BMI, body mass index; BP, blood pressure; CCB, calcium channel blocker; CKD, chronic kidney disease; CK-MB, creatinine kinase-myocardial band; CVA, cerebrovascular accident; DM, diabetes mellitus; DTB, door to balloon; HDL, high-density lipoprotein; HR, heart rate; LDL, low-density lipoprotein; LVEF, left ventricular ejection fraction; MI, myocardial infarction; NSTEMI, non-ST-segment elevation myocardial infarction; NT-proBNP, N-terminal pro-B-type natriuretic peptide, RAS, renin–angiotensin system; STD, symptom to door; STEMI, ST-segment elevation myocardial infarction.

^a^ Fisher's exact test.

### Angiographic and procedural characteristics

The angiographic and procedural characteristics are presented in Table 2. The lower the LVEF, the higher the prevalence of multivessel disease (MVD) (62.8% vs. 49.5% vs. 48.0%; *p* < 0.0001). The patients with reduced and mildly reduced EF frequently presented with culprit lesions in the left anterior descending coronary artery (LAD) at rates of 60.5% and 60.3%, respectively. However, the patients with preserved EF exhibited culprit lesions more commonly in the right coronary artery (RCA) (40.3%), followed by the LAD (38.1%). The proportion of pre-thrombolysis in myocardial infarction (TIMI) grade flow 0 or 1 in patients with mildly reduced EF was the highest among the three groups (63.5% vs. 66.4% vs. 54.7%; *p* < 0.0001). The proportion of post-TIMI grade flow 3 was different among the three groups, despite only a slight difference (94.6% vs. 96.6% vs. 97.4%; *p* < 0.0001). The mildly reduced EF group had an intermediate symptom-to-door (STD) time (300 vs. 221 vs. 92 min; *p* < 0.0001). However, door-to-balloon time (DTB) was the lowest in the mildly reduced EF group (85 vs. 75 vs. 136 min; *p* < 0.0001).

**Table 2:** Angiographic and procedural characteristics.

	Total (*N* = 6,553)	Reduced EF (*n* = 884)	Mildly reduced EF (*n* = 1,749)	Preserved EF (*n* = 3,920)	*p*-value
Disease extent					<0.0001
One-vessel disease	3,253 (49.6)	329 (37.2)	884 (50.5)	2,040 (52.0)	
Two-vessel disease	2,191 (33.4)	335 (37.9)	588 (33.6)	1,268 (32.4)	
Three-vessel disease	1,109 (16.8)	220 (24.9)	277 (15.8)	612 (15.6)	
Multivessel disease	3,300 (50.4)	555 (62.8)	865 (49.5)	1,880 (48.0)	<0.0001
Culprit lesion					<0.0001
Left main	127 (1.9)	47 (5.3)	27 (1.5)	53 (1.4)	
LAD	3,083 (47.1)	535 (60.5)	1,055 (60.3)	1,493 (38.1)	
LCX	1,115 (17.0)	91 (10.3)	229 (13.1)	795 (20.3)	
RCA	2,228 (34.0)	211 (23.9)	438 (25.0)	1,579 (40.3)	
Lesion type					<0.0001
A	46 (0.7)	4 (0.5)	11 (0.6)	31 (0.8)	
B1	801 (12.2)	85 (9.6)	212 (12.1)	504 (12.9)	
B2	2,445 (37.3)	319 (36.1)	587 (33.6)	1,539 (39.3)	
C	3,261 (49.8)	476 (53.9)	939 (53.7)	1,846 (47.1)	
Lesion treatment					0.2107
Stent	6,095 (93.0)	806 (91.2)	1,630 (93.2)	3,659 (93.3)	
Balloon angioplasty	428 (6.5)	74 (8.4)	110 (6.3)	244 (6.2)	
Total number of stents	1.5 ± 0.8	1.6 ± 0.8	1.5 ± 0.8	1.5 ± 0.8	0.0010^a^
GP IIb/IIIa inhibitor	872 (13.3)	109 (12.3)	247 (14.1)	516 (13.2)	0.4047
Thrombolysis	1,876 (28.6)	233 (26.4)	590 (33.7)	1,053 (26.9)	<0.0001
Pre-TIMI					<0.0001
0	3,081 (47.0)	443 (50.1)	940 (53.7)	1,698 (43.3)	
1	784 (12.0)	118 (13.4)	221 (12.6)	445 (11.4)	
2	965 (14.7)	135 (15.3)	225 (12.9)	605 (15.4)	
3	1,723 (26.3)	188 (21.3)	363 (20.8)	1,172 (29.9)	
Pre-TIMI 0, 1	3,865 (59.0)	561 (63.5)	1,161 (66.4)	2,143 (54.7)	<0.0001
Pre-TIMI 3	1,723 (26.3)	188 (21.3)	363 (20.8)	1,172 (29.9)	<0.0001
Post-TIMI					0.0014^b^
0	17 (0.3)	5 (0.6)	5 (0.3)	7 (0.2)	
1	20 (0.3)	4 (0.5)	8 (0.5)	8 (0.2)	
2	171 (2.6)	39 (4.4)	46 (2.6)	86 (2.2)	
3	6,345 (96.8)	836 (94.6)	1,690 (96.6)	3,819 (97.4)	
Post-TIMI 0, 1	37 (0.6)	9 (1.0)	13 (0.7)	15 (0.4)	0.0379
Post-TIMI 3	6,345 (96.8)	836 (94.6)	1,690 (96.6)	3,819 (97.4)	<0.0001

Values are *n* (%) or means ± standard deviations. EF, ejection fraction; GP, glycoprotein; LAD, left anterior descending artery; LCX, left circumflex artery; RCA, right coronary artery; TIMI, thrombolysis in myocardial infarction.

^a^ One-way ANOVA.

^b^ Fisher's exact test.

### Clinical outcomes

**Figure 2:**
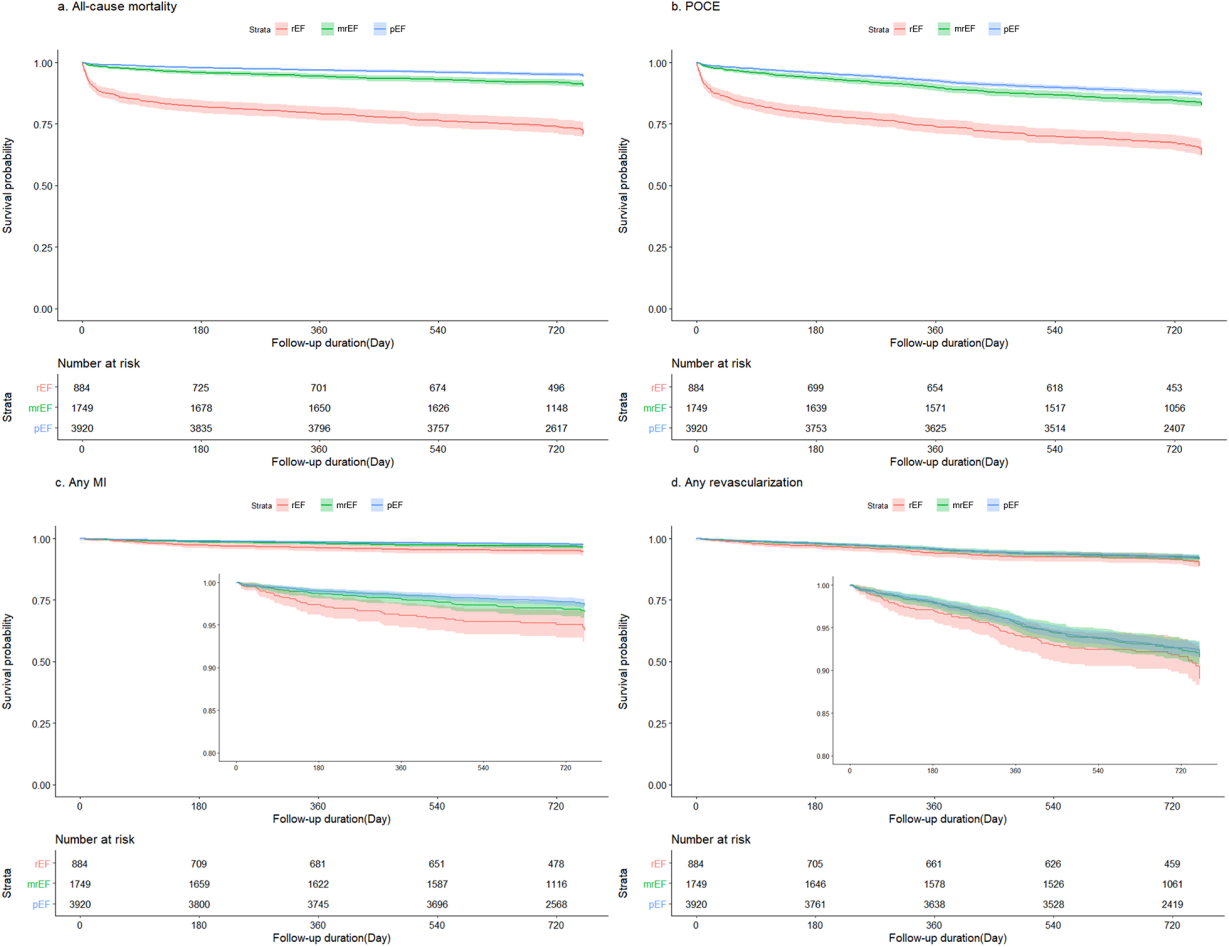
Kaplan–Meier survival curves stratified according to LVEF. LVEF, left ventricular ejection fraction. **(A)** all-cause mortality, **(B)** POCE, **(C)** any MI, and **(D)** any revascularization. MI, myocardial infarction; mrEF, mildly reduced ejection fraction; pEF, preserved ejection fraction; POCE, patient-oriented composite endpoint; rEF, reduced ejection fraction.

Table 3 and Figure 2 summarize the 2-year clinical outcomes of patients after AMI according to the LVEF. The incidence of the primary outcome was intermediate in the mildly reduced EF group (reduced EF, 24.7%; mildly reduced EF, 8.3%; preserved EF, 4.6%; *p* < 0.0001) but close to that in the preserved EF group as was the incidence of MI (3.9% vs. 2.7% vs. 2.6%; *p* = 0.0046) and POCE (33.0% vs. 15.6% vs. 12.4%; *p* < 0.0001). However, the revascularization rate was not significantly different among the three groups (8.3% vs. 7.3% vs. 7.6%; *p* = 0.9339).

**Table 3:** Two-year clinical outcomes according to LVEF.

	Total (*N* = 6,553)	Reduced EF (*n* = 884)	Mildly reduced EF (*n* = 1,749)	Preserved EF (*n* = 3,920)	*p*-value
Primary outcome					
All-cause death	843 (8.0)	314 (24.7)	229 (8.3)	300 (4.6)	<0.0001
Secondary outcomes					
Any MI	290 (2.8)	50 (3.9)	74 (2.7)	166 (2.6)	0.0046
Any revascularization	803 (7.6)	106 (8.3)	200 (7.3)	497 (7.6)	0.9339
POCE	1,654 (15.7)	420 (33.0)	427 (15.6)	807 (12.4)	<0.0001

Values are *n* (%). EF, ejection fraction; LVEF, left ventricular ejection fraction; MI, myocardial infarction; POCE, patient-oriented composite endpoint.

The prognostic factors according to LVEF are summarized in Table 4. Older age, female sex, and low BMI adversely affected the clinical outcomes. Comorbidities, including DM and CKD, were identified as poor prognostic factors in all three groups. A history of MI and CVA was associated with adverse clinical outcomes in the mildly reduced and preserved EF groups, but not in the reduced EF group. A diagnosis of NSTEMI on admission, high heart rate, elevated NT-proBNP level, and low hemoglobin level were significant factors for poor prognosis. The prescription of beta-blockers, RAS inhibitors, and statins at discharge had a significantly beneficial effect on prognosis.

**Table 4:** Predictors for all-cause death according to LVEF.

	Reduced EF (*n* = 884)		Mildly reduced EF (*n* = 1,749)		Preserved EF (*n* = 3,920)	
	HR (95% CI)	*p*-value	HR (95% CI)	*p*-value	HR (95% CI)	*p*-value
Anthropometric data						
Age (per year)	1.06 (1.04–1.07)	<0.0001	1.09 (1.07–1.11)	<0.0001	1.10 (1.08–1.11)	<0.0001
Male	0.74 (0.57–0.96)	0.0213	0.57 (0.41–0.79)	0.0008	0.40 (0.30–0.53)	<0.0001
BMI (per 1 kg/m^2^)	0.96 (0.92–0.99)	0.0206	0.87 (0.84–0.91)	<0.0001	0.85 (0.82–0.88)	<0.0001
Medical history						
Hypertension	1.24 (0.92–1.68)	0.1563	1.92 (1.38–2.67)	<0.0001	0.95 (0.95–1.66)	0.1052
DM	1.74 (1.32–2.30)	0.0001	1.41 (1.01–1.97)	0.0448	1.82 (1.37–2.42)	<0.0001
CKD	2.89 (2.16–3.87)	<0.0001	3.60 (2.62–4.95)	<0.0001	4.50 (3.41–5.93)	<0.0001
Dyslipidemia	0.78 (0.47–1.30)	0.3454	0.55 (0.27–1.13)	0.1035	0.28 (0.13–0.59)	0.0008
Previous MI	1.07 (0.74–1.55)	0.7295	1.73 (1.09–2.75)	0.0193	1.81 (1.10–2.98)	0.0191
Old CVA	1.37 (0.94–2.01)	0.1044	2.39 (1.52–3.76)	0.0002	2.46 (1.61–3.77)	<0.0001
Admission status						
STEMI	0.65 (0.50–0.84)	0.0008	0.45 (0.33–0.62)	<0.0001	0.71 (0.53–0.94)	0.0186
Systolic BP	0.99 (0.99–0.99)	<0.0001	0.99 (0.99–1.00)	0.0028	1.00 (0.99–1.00)	0.3485
Heart rate	1.01 (1.00–1.01)	0.0124	1.01 (1.01–1.02)	0.0008	1.01 (1.00–1.02)	0.0020
Log NT-proBNP	1.44 (1.33–1.56)	<0.0001	1.64 (1.50–1.80)	<0.0001	1.85 (1.72–2.00)	<0.0001
Hemoglobin	0.82 (0.78–0.86)	<0.0001	0.70 (0.60–0.75)	<0.0001	0.69 (0.65–0.73)	<0.0001
Discharge medications						
Beta-blocker	0.20 (0.15–0.26)	<0.0001	0.28 (0.20–0.40)	<0.0001	0.39 (0.28–0.53)	<0.0001
RAS inhibitor	0.26 (0.20–0.33)	<0.0001	0.45 (0.33–0.63)	<0.0001	0.37 (0.28–0.50)	<0.0001
Statin	0.16 (0.12–0.20)	<0.0001	0.27 (0.18–0.40)	<0.0001	0.19 (0.13–0.28)	<0.0001

BMI, body mass index; BP, blood pressure; CI, confidence interval; CKD, chronic kidney disease; CVA, cerebrovascular accident; DM, diabetes mellitus; EF, ejection fraction; HR, hazard ratio; LVEF, left ventricular ejection fraction; MI, myocardial infarction; NT-proBNP, N-terminal pro-B-type natriuretic peptide; RAS, renin–angiotensin system; STEMI, ST-segment elevation myocardial infarction.

In the multivariable Cox proportional hazards model for the primary outcome, the mildly reduced EF group consistently had a better prognosis than the reduced EF group, after adjusting for age, sex, clinical risk factors (DM and CKD), admission status (STEMI, HR, NT-proBNP, and hemoglobin), and discharge medications (beta-blockers, RAS inhibitors, and statins). However, as compared to the preserved EF group, there were no significant differences in primary outcome (Table 5).

**Table 5:** Multivariable Cox proportional hazard model for all-cause death.

	Reduced EF	Mildly reduced EF	Preserved EF	Adjusted p* (mildly reduced vs. reduced EF)	Adjusted p* (mildly reduced vs. preserved EF)
	HR (95% CI)		HR (95% CI)		
Model 1	3.03 (2.17–3.71)	1	0.65 (0.53–0.81)	<0.001	0.150
Model 2	2.59 (2.09–3.20)	1	0.69 (0.56–0.86)	<0.001	0.270
Model 3	1.81 (1.46–2.26)	1	0.91 (0.72–1.13)	<0.001	0.999
Model 4	1.74 (1.40–2.18)	1	0.94 (0.75–1.18)	<0.001	0.999

Model 1; age, sex. Model 2; Model 1 + clinical risk factors (DM, CKD). Model 3; Model 2 + admission status (STEMI, heart rate, NT-proBNP, hemoglobin). Model 4; model 3 + discharge medications (beta-blocker, RAS inhibitor, statin). CI, confidence interval; CKD, chronic kidney disease; DM, diabetes mellitus; EF, ejection fraction; HR, hazard ratio; NT-proBNP, N-terminal pro-B-type natriuretic peptide; RAS, renin–angiotensin system; STEMI, ST-segment elevation myocardial infarction.

* *Post hoc* analysis was conducted based on Bonferroni correction.

## Discussion

We investigated the clinical characteristics and long-term prognoses of patients with mildly reduced EF after AMI. The key findings are as follows. First, patients with a mildly reduced EF showed intermediate clinical features. However, the prevalence of comorbidities in the mildly reduced EF group tended to be closer to that in the preserved EF group. Second, the angiographic and procedural characteristics of the mildly reduced EF group were mostly similar to those of the reduced EF group. The proportion of culprit lesion locations (LAD) and pre-TIMI grade flow 0 or 1 were comparable between the mildly reduced and reduced EF groups, which are significantly higher than in the preserved EF group. However, the proportion of MVD was lower in the mildly reduced HF group than in the reduced EF group. Finally, the mildly reduced EF group had an intermediate prognosis among the three groups but was similar to the preserved EF group.

### Clinical and procedural characteristics of patients with mildly reduced EF after AMI

In HF, HFmrEF has been reported to have similar features to HFrEF in terms of a younger age, male predominance, a high incidence of IHD, and comorbidities such as DM and CKD. However, HFmrEF has been reported to have similar features to HFpEF in terms of older age, BMI, comorbidities (e.g., HTN and atrial fibrillation), and laboratory markers (e.g., natriuretic peptide levels) (12, 15, 19, 24). Cho et al. (25) also reported that HFmrEF exhibited intermediate features, and IHD was the predominant etiology in the HFmrEF group. However, a detailed analysis of the clinical presentation and procedural aspects specific to patients with IHD has not been conducted. In this study, we analyzed not only the clinical factors but also the procedural factors and medication usage in patients with AMI according to the EF spectrum.

Our study revealed an inverse distribution of demographic and clinical risk factors compared to previous HF studies. The preserved EF group was the youngest and had the highest proportion of male patients. Additionally, each of the three groups exhibited a notable predominance of male patients. The comorbidities also showed distinct patterns. Although the prevalence of HTN in the mildly reduced EF group was similar to that in the preserved EF group, the prevalence rates of DM and CKD were similar to those in the reduced EF group. NT-proBNP levels exhibited intermediate values but were much closer to those of the preserved EF group. The prescriptions for discharge medication also showed a different trend when compared to those prescribed to HF populations. Cho et al. (25) reported that guideline-directed medical therapy maintenance was more prevalent in patients with HFrEF. However, in our study, patients with reduced EF were prescribed the fewest medications. In angiographic and procedural factors, patients with mildly reduced EF had a comparable proportion of STEMI to those with reduced EF. These two groups were also similar in terms of culprit lesion predominance in the LAD and reduced pre-TIMI grade flow. However, the proportion of MVD in the mildly reduced EF group was significantly lower than in the reduced EF group.

### Prognosis of patients with mildly reduced EF after AMI

In HF, HFmrEF has shown an intermediate risk of overall mortality, adverse cardiovascular events, and non-cardiovascular outcomes (14, 15). Non-cardiovascular events were more prevalent in the HFpEF, characterized by higher comorbidity burdens and older age (14, 15). Conversely, cardiovascular events were the most pronounced in the HFrEF, reflecting a higher proportion of IHD (24). In randomized trials, patients with HFmrEF have shown similar rates of first HF hospitalization, cardiovascular death, and all-cause mortality compared to those with HFpEF, all of which were notably lower than those observed in patients with HFrEF (19, 26). Cho et al. reported that 3-year all-cause mortality in patients with HF did not differ significantly by EF spectrum. However, unlike non-ischemic HF, patients with ischemic HF showed differences in 3-year all-cause mortality based on EF. In particular, only the ischemic etiology has emerged as the strongest risk factor for in-hospital death in HFmrEF (25).

In the present study, patients with mildly reduced EF after AMI exhibited intermediate adverse outcomes. As the LVEF decreased, the age, number of comorbidities, and Killip class tended to increase, all of which contributed to a poor prognosis. However, the disparity in clinical outcomes between the mildly reduced and preserved EF groups was not statistically significant. Interestingly, despite the similarities of the angiographic and procedural characteristics between the mildly reduced EF and reduced EF groups, prognosis differed significantly between these two groups. This discrepancy was attributed to the lower prevalence of MVD in the mildly reduced EF group than in the reduced EF group. Additionally, the reduced EF group had a significantly lower rate of medication use at discharge than the other two groups. This disparity is likely attributable to the increased prevalence of comorbidities in the reduced EF group, which included more hemodynamically compromised patients presenting with a Killip class of ≥II. This may have hindered the prescription of medications and could have impacted the prognosis.

**Figure 3:**
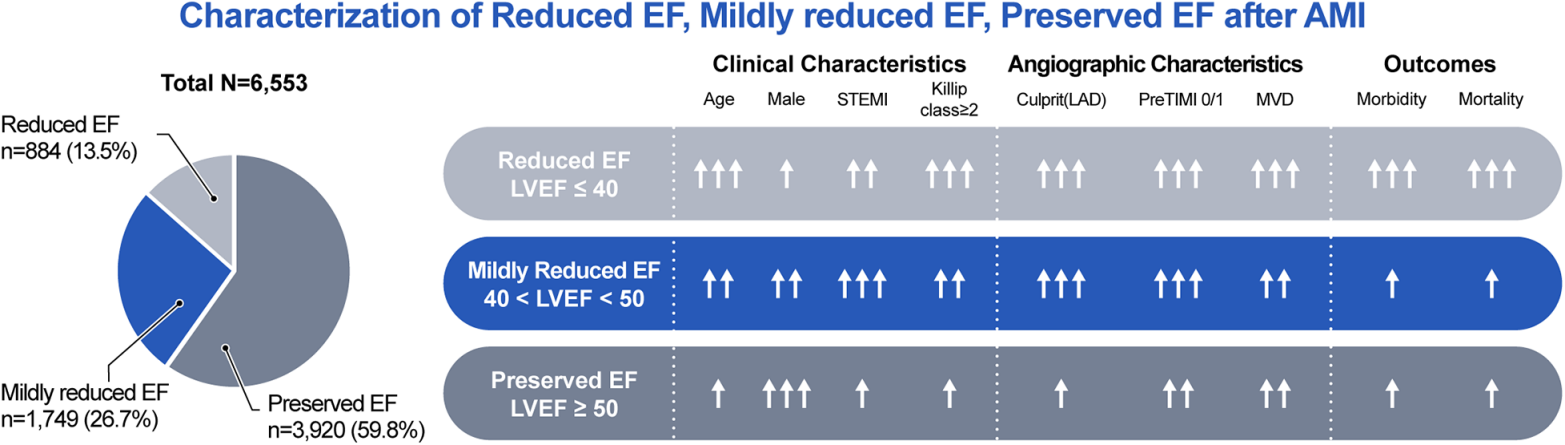
Characterization of reduced EF, mildly reduced EF, preserved EF after AMI. AMI, acute myocardial infarction; EF, ejection fraction; LAD, left anterior descending artery; LVEF, left ventricular ejection fraction; MVD, multivessel disease; STEMI, ST-segment elevation myocardial infarction; TIMI, thrombolysis in myocardial infarction.

The degree of neurohormonal activation, as indicated by biomarkers such as BNP or NT-proBNP, may serve as a marker of the severity of HF and a high incidence of cardiovascular events. Subsequently, the degree of neurohormonal suppression may be considerable in HF (27–30). Nevertheless, several trials have demonstrated that RAS inhibitors, beta-blockers, mineralocorticoid receptor antagonists (MRAs), and angiotensin receptor neprilysin inhibitors (ARNIs) are ineffective in HFpEF, with an EF of ≥40 or 45% (29–33). In a previous study from the Korean AMI Registry, beta-blockers were associated with reduced or tended to reduce 2-year all-cause mortality in patients with reduced EF or mildly reduced EF, but not in those with preserved EF (20). In another study from the Korean AMI Registry, beta-blockers or RAS inhibitors at discharge were associated with improved 2-year clinical outcomes without a significant difference between an EF of ≤45% and >45% in patients with mildly reduced EF (34). Recently, in the Randomized Evaluation of Decreased Usage of Beta-Blockers after Acute Myocardial Infarction (REDUCE-AMI) trial, long-term beta-blocker treatment in patients with preserved EF did not lead to a lower risk of death from any cause or new MI than no beta-blocker use (35). In our study, the use of RAS inhibitors, beta-blockers, and statins at discharge is a protective factor for primary outcomes regardless of EF. The clinical, angiographic, and outcomes for each group are summarized in Figure 3.

### Limitations

This study had some limitations. First, this study was a prospective, observational multicenter registry. Therefore, the possibility of an inherent selection bias cannot be ignored. Second, we classified patients based solely on the EF spectrum. However, there are many ways to assess infarct size and prognosis in patients with AMI beyond the simple measurement of EF alone. Speckle tracking parameters on echocardiography can be used to predict left ventricular remodeling and prognosis (36). Furthermore, quantitative factors such as infarct size, extent of viability, and edema in non-infarct territory on cardiac magnetic resonance imaging (MRI) can be used to assess prognosis in patients with AMI (37). However, the lack of available data precluded the performance of the requisite analysis in this study. Third, information on the occurrence of HF and the prescription of medications, such as MRAs, ARNIs, or sodium–glucose cotransporter 2 (SGL2) inhibitors, was lacking. However, in the Prospective ARNI vs. ACE Inhibitor Trial to Determine Superiority in Reducing Heart Failure Events after Myocardial Infarction (PARADISE-MI), ARNI was not associated with a lower incidence of death from cardiovascular causes or incident HF than ramipril among patients with AMI. In this trial, participants had LVEF below 40%, and more than half of them had pulmonary congestion (38). In the Study to Evaluate the Effect of Empagliflozin on Hospitalization for Heart Failure and Mortality in Patients with Acute Myocardial Infarction (EMPACT-MI), 78.4% of participants had LVEF of <45%, and 57.0% of participants had signs or symptoms of congestion. In this trial, empagliflozin did not reduce the risk of the composite outcome of first hospitalization for HF or death from any cause than placebo (39). Fourth, while discharge medication prescriptions were analyzed, long-term adherence could not be investigated. Fifth, the study did not include information on ECG findings, such as atrial fibrillation and left bundle branch block, both of which are significant hemodynamic factors. Finally, the transition of patients from a mildly reduced EF group to an improved or persistent EF group was unclear. In HF, HFmrEF often transitions dynamically to HFpEF or HFrEF, particularly within a year. This suggests that HFmrEF may represent a transitional state or an overlapping zone between HFpEF and HFrEF rather than an independent entity (27). Therefore, a comprehensive assessment of EF trajectories in patients with AMI is also needed.

## Conclusion

In conclusion, this study elucidated the distinct clinical features and prognosis of patients with mildly reduced EF after AMI. These patients showed intermediate demographic and clinical factors. The angiographic and procedural characteristics of the mildly reduced EF group were comparable with those of the reduced EF group, except for the extent of MVD. Overall, patients with mildly reduced EF showed a better prognosis than those with reduced EF and similar to those with preserved EF.

## Data Availability

The original contributions presented in the study are included in the article; further inquiries can be directed to the corresponding author.
